# A better prediction of progression‐free survival in diffuse large B‐cell lymphoma by a prognostic model consisting of baseline TLG and %ΔSUV_max_

**DOI:** 10.1002/cam4.2284

**Published:** 2019-07-25

**Authors:** Yi‐Yang Zhang, Le Song, Mei‐Xin Zhao, Kai Hu

**Affiliations:** ^1^ Department of Hematology Peking University Third Hospital Beijing China; ^2^ Department of Nuclear Medicine Peking University Third Hospital Beijing China

**Keywords:** diffuse large B‐cell lymphoma, metabolic tumor volume, prognostic model, total lesion glycolysis

## Abstract

In the era of rituximab, the International Prognostic Index (IPI) has been inefficient in initial risk stratification for patients with R‐CHOP‐treated diffuse large B‐cell lymphoma (DLBCL). To estimate the predictive values of PET/CT quantitative parameters and three prognostic models consisting of baseline and interim parameters for three‐year progression‐free survival (PFS), we conducted an analysis of 85 patients in China with DLBCL underwent baseline and interim PET/CT scans and treated at the Department of Hematology of Peking University Third Hospital from November 2012 to November 2017. The PET/CT parameters, viz. the baseline and interim values of standardized uptake value (SUV_max_), total metabolic tumor volume (TMTV), and total lesion glycolysis (TLG), and their rates of change, were analyzed by a receiver operating characteristics curve, Kaplan‐Meier analysis, and log‐rank test. Besides, the National Comprehensive Cancer Network International Prognostic Index (NCCN‐IPI) was also included in the multivariate Cox hazards model. Owing to the strong correlation between TMTV and TLG at baseline and interim (Pearson's correlation coefficient, *r* = 0.823, *P‐*value* = *0.000, and 0.988, *P‐*value = 0.000, respectively), only TLG was included in the multivariate Cox hazards model, where TLG_0_ > 1036.61 g and %ΔSUV_max_ < 86.02% showed predictive value independently (HR = 10.42, 95% CI 2.35‐46.30, *P = *0.002, and HR = 4.86, 95% CI 1.27‐18.54, *P = *0.021, respectively). Replacing TLG in the equation, TMTV_0_ and TMTV_1_ both showed significantly predictive abilities like TLG (HR = 8.22, 95% CI 1.86‐32.24, *P = *0.005, and HR = 2.96, 95% CI 1.16‐7.54, *P = *0.023, respectively). After dichotomy, NCCN‐IPI also gave a significant performance (*P* = 0.035 and *P* = 0.010, respectively, in TLG and TMTV models). The baseline variables, that is, TMTV_0_, TLG_0_ and dichotomized NCCN‐IPI, and the interim variables TMTV_1_ and %ΔSUV_max_, presented independent prognostic value for PFS. In prognostic model 2 (TLG_0_ + %ΔSUV_max_), the group with TLG_0_ > 1036.61 g and %ΔSUV_max_ < 86.02% recognized 19 (82.6%) of the relapse or progression events, which showed the best screening ability among three models consisting of baseline and interim PET/CT parameters.

## 1 INTRODUCTION

Diffuse large B‐cell lymphoma (DLBCL) is the most prevalent type of non‐Hodgkin lymphoma. Although the addition of rituximab to a CHOP (cyclophosphamide, doxorubicin, vincristine, and prednisone)‐like regimen (R‐CHOP) has improved DLBCL outcomes significantly, over 25% of patients treated with R‐CHOP unfortunately experience treatment failure.^1^ The early recognition of patients with a poor prognosis and the tailoring of their curative remediation plan are undoubtedly key interventions. For the past 20 years, the International Prognostic Index (IPI) has been the basis for initial risk stratification for patients with CHOP‐treated DLBCL, facilitating treatment selection and prognosis evaluation. However, the advent of rituximab reduced the prognostic ability of the IPI. In 2013,^2^ National Comprehensive Cancer Network International Prognostic Index (NCCN‐IPI), an enhanced IPI, was recommended to discriminate the high‐risk group, which was also demonstrated in eastern ethnic populations.^3^, ^4^ However, after evaluating the NCCN‐IPI in 284 Japanese patients with R‐CHOP‐treated DLBCL, Nakaya et al^5^ concluded that this index did not reflect progression‐free survival (PFS) in their cohort. Adams and Kwee^6^ thought that patients with a high‐risk NCCN‐IPI still had quite a high PFS rate of 40‐60%. Another effective method was to evaluate quantitative parameters derived from F^18^‐fluorodeoxyglucose positron emission tomography‐computed tomography (^18^F‐FDG PET/CT). PET/CT has been introduced into the guidelines of the National Comprehensive Cancer Network because of its capabilities of accurately revealing the stages of cancers and monitoring the effects of therapies. Among several parameters of PET/CT, standardized uptake value (SUV_max_) was the most common for quantifying tracer uptake. SUV‐related quantitative measures, such as total metabolic tumor volume (TMTV) and total lesion glycolysis (TLG), which can assess the baseline and interim tumor burden, have gained increasing importance for therapy response monitoring and prognostic assessment.^7^ However, interpretations of these parameters are still controversial. Owing to the unstable manifestations of assessments and prognostic values reported in many studies,^8^, ^9^, ^10^, ^11^, ^12^, ^13^ the interests of researchers have gradually moved toward the TMTV and TLG parameters. Interim PET/CT parameters have demonstrated prognostic value in Hodgkin lymphoma, and several studies are testing the response‐adapted treatment regimens. If these interim parameters have the same role in DLBCL, we would also be able to try clinical trials of response‐adapted treatment regimens for DLBCL. Thus, more studies of the quantitative parameters are needed to assess their ability in discriminating high‐risk patients, and to compare the superiority of PET/CT and the NCCN‐IPI.

This study sought to retrospectively analyze the association between relapsed/refractory disease and the clinical characteristics, NCCN‐IPI, and PET/CT‐related quantitative parameters (baseline and interim SUV_max_, TMTV, and TLG), and to explore new prognostic models that combines baseline and interim parameters for discriminating high‐risk patients efficiently.

## 2 MATERIALS AND METHODS

### 2.1 Subjects

A retrospective study of 85 consecutive patients newly histologically diagnosed with DLBCL was performed. All of them had undergone a baseline PET/CT scan before initial R‐CHOP or R‐CHOP‐like therapy and an interim PET/CT scan after 2‐4 cycles of chemotherapy at the Department of Hematology of Peking University Third Hospital from November 2012 to November 2017. Inclusion criteria were as follows: (a) age ≥18 years; (b) histologically confirmed DLBCL; (c) treated with R‐CHOP or R‐CHOP‐like chemotherapy; (d) completed baseline and interim PET/CT scans; and (e) with complete clinical information. The following were the exclusion criteria: (a) presence of concurrent acute or chronic infections; (b) malignant tumor history; and (c) lactating or pregnant. The Medical Research Ethics Committee of Peking University Third Hospital approved the study procedures. Informed consents were obtained from all of the patients, who were informed that the study would be conducted anonymously and their privacy would thus be respected.

The clinical information consisted of the patient's age, gender, B symptom, Ann Arbor stage, Eastern Cooperative Oncology Group performance status (ECOG PS), lactate dehydrogenase (LDH) ratio, extranodal disease, NCCN‐IPI score and risk groups, baseline and interim quantitative parameters (SUV_max_, TMTV, and TLG), therapeutic regimen, follow‐up time, current status, and PFS. PFS was defined as the first date of documentation of a new lesion or enlargement of a previous lesion, or death from the disease.^14^ Based on The Lugano Classification,^15^ progressive metabolic disease was defined as PET/CT score 4 or 5 with an increase in intensity of uptake from baseline and/or new FDG‐avid foci consistent with lymphoma at interim PET/CT assessment. The NCCN‐IPI score used a maximum of eight points for the categorized age (41‐60 years, 1 point;61‐75 years, 2 points; >75 years, 3 points) and LDH ratio (1‐3 times, 1 point; >3 times, 2 points) at the upper limit of normal, in addition to extranodal disease involvement in major organs (bone marrow, central nervous system, liver/gastrointestinal tract, or lung), Ann Arbor stage III/IV, and ECOG PS (≥2), each carrying 1 point. PFS was defined as lymphoma progression or death as a result of any cause measured from the time point of entry into the study.

### 2.2 ^18^F‐FDG PET/CT

All the data were acquired and processed with the Siemens 52‐cycles Biograph 64 PET/CT scanner and MedEx PET/CT central imaging and information system, respectively. ^18^F‐FDG was supplied by the Institute of Isotope, China Institute of Atomic Energy Sciences. Before FDG injection, patients rested for at least 6 hours without parenteral nutrition and the serum glucose level was decreased to the normal levels (typically 4‐7 mmol/L). After injection of the 0.10‐0.15 mCi/kg ^18^F‐FDG, the patients rested for 60 minutes before the PET/CT scan. The PET images were collected by scanning 5‐7 bed positions (2.0‐minute acquisition time per bed position), covering the region from the base of the skull through to the upper thigh. PET images were reconstructed with TrueX algorithm, Iterations 4, Subsets 16, Zoom 2.7, FWHM 4.0. The final images were evaluated with the MedEx PET/CT central imaging and information system. The baseline PET/CT scan was obtained before initial R‐CHOP treatment, and the interim scan was completed after 2‐4 chemotherapy cycles.

### 2.3 Image evaluation

The evaluations of both the baseline and interim images were completed by two senior nuclear medicine radiologists, respectively, using the MedEx PET/CT central imaging and information system. The general definition of a positive (abnormal) PET finding (using visual assessment) as being a focal or diffuse FDG uptake above background in a location incompatible with normal anatomy/physiology seems to be appropriate in the majority of cases. However, the following exceptions were noted^16^, ^17^: (a) mild and diffusely increased FDG uptake at the site of moderate‐sized or large residual masses (ie ≥2 cm in diameter), with an intensity no more than that of the mediastinal blood pool (MBP), was considered negative for the presence of residual lymphoma, whereas diffuse or focal uptake exceeding that of the MBP was considered indicative of lymphoma; (b) new lung nodules ≥1.5 cm in patients with no evidence of pulmonary lymphoma before therapy were considered suggestive of lymphoma if their uptake exceeded that of the MBP, whereas the degree of uptake was unreliable for assessment for nodules <1.5 cm owing to partial volume averaging; and (c) clearly increased (multi)focal bone (marrow) uptake was considered positive for lymphoma, whereas diffusely increased FDG uptake in the bone marrow at 2‐3 weeks after chemotherapy was not misinterpreted as diffuse lymphomatous marrow involvement.

With the fixed threshold method (41% of focal lesion SUV_max_), we delineated the region of interest around the focus lesions. The system semi‐automatically collected, processed, and output the SUV_max_, SUV_mean_, and TMTV data. Whole‐body TLG was calculated as Σ(SUV_mean_ × MTV). Baseline PET/CT parameters were recorded as SUV_max0_, TMTV_0_, and TLG_0_, following which the interim parameters, difference, and difference ratio were recorded, respectively, as SUV_max1_, TMTV_1_, and TLG_1_; ΔSUV_max_, ΔTMTV, and ΔTLG; and %ΔSUV_max_, %ΔTMTV, and %ΔTLG.

### 2.4 Statistical analysis

The NCCN‐IPI scores were categorized into four risk groups: low (0‐1), low‐intermediate (2‐3), high‐intermediate (4‐5), and high (6‐8). The baseline and interim parameters were combined for prognosis analysis. Descriptive analysis and chi‐squared tests were applied for the clinical information and their relationship with PFS. A receiver operating characteristic (ROC) curve was used to determine the optimal cutoff values of SUV_max_, TMTV, and TLG for 3‐year % PFS, where the cutoff values of these parameters with AUC > 0.7 were determined by the Youden index (maximum sum of sensitivity and specificity). Survival analysis was completed with a Kaplan‐Meier (K‐M) survival analysis, and differences between groups were analyzed with the log‐rank test. Independent predictive variables were determined with univariate and multivariate Cox regression analyses by the method of Forward LR Pearson's correlation coefficient analysis was used for the bivariate correlation analysis of two likely correlated parameters (eg TMTV and TLG). All analyses were conducted using SPSS 19.0 (IBM, Armonk, NY, USA). A two‐sided *P*‐value of less than 0.05 was considered significant.

## 3 RESULTS

### 3.1 Clinical characteristics

The age and three‐year PFS distribution for all the patients are summarized in Table 1. There were a total of 85 enrolled patients (46 men, 39 women, age 55.4 ± 16 years). The median follow‐up time was 34 months (range: 7‐76 months). In total, 62 (72.9%) patients survived for 3 years without disease progression or relapse or death from the disease. Twenty‐three (27.1%) encountered disease progression or relapse at a median of 14.6 months. Patients with a B symptom, higher Ann Arbor stage, higher LDH ratio, extranodal disease involvement in major organs, and higher NCCN‐IPI risk showed a significantly lower three‐year PFS.

**Table 1:** Clinical information and three‐year PFS for the study participants

Characteristic		Total (%)	3‐Year PFS, N (%)	Chi‐square value	*P*‐Value
Age, years	>18 to ≤40	15 (17.6)	13 (86.7%)	6.335	0.096
	>41 to ≤60	36 (42.4)	29 (80.6%)		
	>61 to ≤75	26 (30.6)	16 (61.5%)		
	>75	8 (9.4)	4 (50.0%)		
Gender	Male	46 (54.1)	32 (69.6%)	0.579	0.447
	Female	39 (45.9)	30 (76.9%)		
B symptoms	No	62 (72.9)	49 (79.0%)	4.307	0.038
	Yes	23 (27.1)	13 (56.5%)		
Ann Arbor stage	I/II	32 (37.6)	29 (90.6%)	8.131	0.004
	III/IV	53 (62.4)	33 (62.3%)		
ECOG PS	0‐1	71 (83.5)	54 (76.1%)	2.119	0.145
	≥2	14 (16.5)	8 (57.1%)		
LDH ratio	≤1	44 (51.8)	37 (84.1%)	6.187	0.045
	>1 to ≤3	37 (43.5)	22 (59.5%)		
	>3	4 (4.7)	3 (75.0%)		
Extranodal disease^a^	No	31 (36.5)	28 (90.3%)	7.469	0.006
	Yes	54 (63.5)	34 (63.0%)		
NCCN‐IPI risk groups	Low (0‐1)	13 (15.3)	12 (92.3%)	18.270	0.000
	Low‐intermediate (2‐3)	36 (42.4)	31 (86.1%)		
	High‐intermediate (4‐5)	25 (29.4)	16 (64.0%)		
	High (6‐8)	11 (12.9)	3 (27.3%)		
Total		85 (100)	62 (72.9%)		

Abbreviations: ECOG PS, Eastern Cooperative Oncology Group performance status; LDH, lactate dehydrogenase, NCCN‐IPI, National Comprehensive Cancer Network International Prognostic Index; PFS, progression‐free survival.

a Disease in the bone marrow, CNS, liver/gastrointestinal tract, or lung.

### 3.2 ROC analysis of quantitative PET/CT parameters

Table 2 summarizes the baseline, interim, difference between them, and difference ratio of the PET/CT quantitative parameters. Parameters with AUCs < 0.7, that is, SUV_max0_, ΔSUV_max_, ΔTMTV, and ΔTLG, were excluded. Then, the cutoff values of the others were calculated by the maximum Youden index. The results of them were as follows: TMTV_0_ (AUC 0.745; cutoff value 80.74 cm^3^; sensitivity/specificity 91.3%/56.5%), TLG_0_ (0.738; 1036.61 g; 91.3%/56.5%), SUV_max1_ (0.751; 3.85; 69.6%/74.2%), TMTV_1_ (0.735; 4.32 cm^3^; 60.9%/87.1%), TLG_1_ (0.737; 14.07 g; 60.9%/85.5%), %ΔSUV_max_ (0.715; 86.02%; 87%/51.6%), %ΔTMTV (0.723; 99.22%; 60.9%/85.5%), and %ΔTLG (0.727; 99.86%; 60.9%/83.9%). TMTV_0_ and TLG_0_ had the best performance in terms of the sensitivity of disease progression or relapse. The %ΔSUV_max_ showed the best sensitivity among the interim parameters. To avoid mistaken eliminations of parameters, we also tested the dichotomized ΔTMTV and ΔTLG for PFS owing to their AUCs being nearly 0.7, but got unsatisfactory results.

**Table 2:** Receiver operating characteristic curve analysis and three‐year PFS from related parameters

Variables	AUC	Cutoff value	Sensitivity (%)	Specificity (%)	Three‐year PFS (above vs. below cutoffs) (%)
SUV_max0_	0.573	–	–	–	–
TMTV_0_	0.745	80.74	91.3	56.5	56.3 vs. 94.6
TLG_0_	0.738	1036.61	91.3	56.5	56.3 vs. 94.6
SUV_max1_	0.751	3.85	69.6	74.2	50.0 vs. 86.8
TMTV_1_	0.735	4.32	60.9	87.1	36.4 vs. 85.7
TLG_1_	0.737	14.07	60.9	85.5	39.1 vs. 85.5
%ΔSUV_max_	0.715	86.02	87	51.6	91.4 vs. 60.0
%ΔTMTV	0.723	99.22	60.9	85.5	85.5 vs. 39.1
%ΔTLG	0.727	99.86	60.9	83.9	85.2 vs. 41.7
ΔSUV_max_	0.446	–	–	–	–
ΔTMTV	0.689	–	–	–	–
ΔTLG	0.683	–	–	–	–
NCCN‐IPI (>3 vs. ≤3)					52.8 vs. 87.8

AUC, area under the receiver operating characteristic curve; MTV, metabolic tumor volume; NCCN‐IPI, National Comprehensive Cancer Network International Prognostic Index; PFS, progression‐free survival; SUV_max_, maximum standardized uptake value; TLG, total lesion glycolysis. The subscripts 0 and 1 represent baseline and interim measures, respectively.

### 3.3 Kaplan‐Meier survival curve and Cox regression analysis

**Figure 1:**
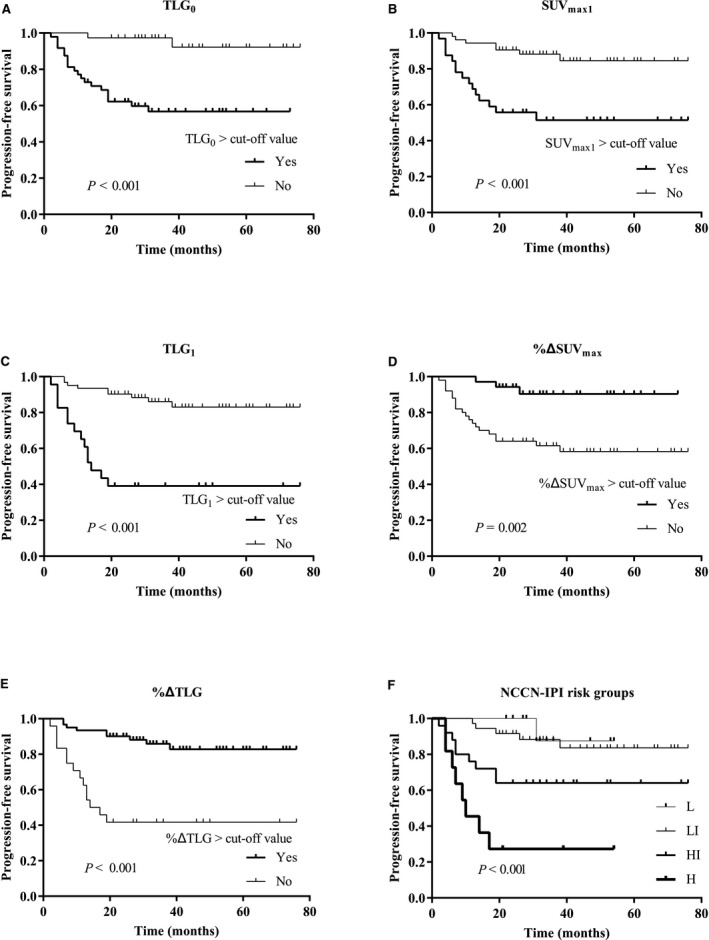
Kaplan‐Meier survival analysis of dichotomized quantitative parameters of PET/CT and NCCN‐IPI risk groups. H, high‐risk group; HI, high‐intermediate risk group; L, low‐risk group; LI, low‐intermediate risk group; NCCN‐IPI, National Comprehensive Cancer Network International Prognostic Index; SUVmax, maximum standardized uptake value; TLG, total lesion glycolysis. The subscripts 0 and 1 represent baseline and interim measures, respectively

As both of the baseline and interim TMTV and TLG values showed a strong correlation (Pearson's correlation coefficient, *r* = 0.823 and 0.988, respectively, and both of *P‐*value* = *0.000) due to the similar calculation mode, TLG reflected the tumor metabolic intensity in addition to the tumor volume, which could possibly be a better estimate of tumor burden compared with the TMTVs. Consequently, only TLGs were included in the K‐M and multivariate Cox regression analysis. In Data S1 section, we can also find those results after TMTV replacing TLG. Based on cutoff values derived from the ROC curve analysis, these dichotomized quantitative variables showed significantly separated survival curves by K‐M analysis (Figure 1). All of the higher groups of TLG_0_, SUV_max1_, and TLG_1_ presented significantly poorer PFS. Patients with %ΔSUV_max_, %ΔTMTV, and %ΔTLG less than the cutoff values got the same poor PFS. In the K‐M analysis of the NCCN‐IPI risk groups, great differences were presented between the low‐ and high‐risk groups, especially for the high‐intermediate and high‐risk groups, but the survival curves for the low‐ and low‐intermediate risk groups almost converged.

The NCCN‐IPI, TMTV_0_, TLG_0_, SUV_max1_, TMTV_1_, TLG_1_, and %ΔSUV_max _all showed significantly predictive values for PFS in the univariate Cox analysis. Due to the strong correlation between TMTV and TLG, only TLGs were included in the multivariate Cox regression analysis. Besides, due to the same correlation between TLGs (or TMTVs) and %ΔTLG(or %ΔTMTV), %ΔTLG and %ΔTMTV were also excluded. Because of the better performance of %ΔSUV_max _in sensitivity derived from ROC analysis, SUV_max1_ strongly correlated with %ΔSUV_max _was also excluded.

Eventually, NCCN‐IPI, TLG_0_, TLG_1_, and %ΔSUV_max _were entered into the multivariate COX regression analysis, but only TLG_0_ and %ΔSUV_max_ showed predictive value independently (hazard ratio, HR = 10.42, 95% confidence interval, CI 2.35‐46.30, *P‐*value* = *0.002, and HR = 4.86, 95% CI 1.27‐18.54, *P = *0.021, respectively). We also conducted another multivariate model analysis by replacing the TLGs with the TMTVs, where TMTV_0_ and TMTV_1_ both showed significantly predictive abilities (Table S1). Although the NCCN‐IPI scores of the four risk groups (low‐, low‐intermediate, high‐intermediate, and high‐risk groups) were initially included in the multivariate analysis, they failed when entered into the model equation (Table 3). However, we also tried analyzing a multivariate regression equation model using the dichotomized NCCN‐IPIs (divided into two groups of low/low‐intermediate and high‐intermediate/high‐risk groups) and concluded that TLG_0_, TLG_1_, and the dichotomized NCCN‐IPIs showed predictive value independently (HR = 5.839, 95% CI 1.20‐28.44, *P*‐value = 0.029; HR = 3.082, 95% CI 1.15‐8.25, *P = *0.025; HR = 3.00, 95% CI 1.08‐8.33, *P = *0.035, respectively) (Table S2), whereas that of %ΔSUV_max_ in this model showed a trend for significance as an independent predictor of PFS (HR = 3.80, 95% CI 0.98‐14.77, *P*‐value = 0.054). TMTVs also presented similar results after replacing TLGs (Table S2).

**Table 3:** Risk factors of clinical and quantitative PET/CT parameters for PFS analyzed by univariate and multivariate Cox regression analyses

Covariate	Univariate analyses			Multivariate analyses		
	HR	95% CI	*P*‐Value	HR	95% CI	*P*‐Value
NCCN‐IPI risk groups	–	–	0.000	–	–	–
Low	–	–	–	–	–	–
Low‐intermediate	1.79	0.21, 15.36	0.59	–	–	–
High‐intermediate	5.72	0.73, 45.2	0.098	–	–	–
High	17.49	2.17, 140.99	0.007	–	–	–
TMTV_0_ (>80.74)	10.32	2.42, 44.084	0.002	–	–	–
TLG_0_ (>1036.61)	10.39	2.43, 44.39	0.002	10.42	2.35, 46.30	0.002
TMTV_1_ (>4.32)	6.93	2.97, 16.17	0.000	–	–	–
TLG_1_ (>14.07)	6.25	2.68, 14.56	0.000	2.39	0.93, 6.16	0.072
%ΔSUV_max_ (<86.02)	5.60	1.66, 18.88	0.005	4.86	1.27, 18.54	0.021

CI, confidence interval; HR, hazards ratio; MTV, metabolic tumor volume; NCCN‐IPI, National Comprehensive Cancer Network International Prognostic Index; PFS, progression‐free survival; SUV_max_, maximum standardized uptake value; TLG, total lesion glycolysis. The subscripts 0 and 1 represent baseline and interim measures, respectively. The MTV variables were not included in the multivariate model owing to their correlation with the TLG variables.

### 3.4 Predictive models

On the basis of the ROC analysis and the multivariate Cox model (Tables 2 and 3), three models consisting of both baseline and interim parameters (viz. model 1[TLG_0_ + TLG_1_], model 2[TLG_0_ + %ΔSUV_max_], and model 3[NCCN‐IPI + %ΔSUV_max_]) were built and analyzed by K‐M analysis and log‐rank test (Table 4). As a result, in model 2, the group with TLG_0_ > 1036.6 and %ΔSUV_max_ < 86.02% recognized 19 (82.6%) of the relapse or progression events, whereas only four events were picked out by the other three risk groups (Table 4). The three‐year PFS of this group was 32.1%, whereas all these of the other three groups were more than 90%. In model 1, the double‐positive group predicted a lower three‐year PFS of 27.8% and picked out 13 of 23 patients with relapse or progression, whereas 10 of them were still omitted in the other three risk groups.

**Table 4:** Results of three prognostic models derived by Kaplan‐Meier survival analysis

Model	Baseline variables	Interim variables	n	Three‐year PFS, N (%)	*P*‐Value^a^
Model 1	TLG_0_ ≤ 1036.6	TLG_1_ ≥ 14.068	32	31 (96.90)	0.157
		TLG_1_ > 14.068	5	4 (80.00)	
		Total	37	35 (94.60)	
	TLG_0_ > 1036.6	TLG_1_ ≤ 14.068	30	22 (73.30)	0.000
		TLG_1_ > 14.068	18	5 (27.80)	
		Total	48	27 (56.30)	
Model 2	TLG_0_ ≤ 1036.6	ΔSUV_max_ ≥ 86.02%	15	14 (93.3)	0.547
		ΔSUV_max_ < 86.02%	22	21 (95.5)	
		Total	37	35 (94.6)	
	TLG_0_ > 1036.6	ΔSUV_max_ ≥ 86.02%	20	18 (90.0)	0.000
		ΔSUV_max_ < 86.02%	28	9 (32.1)	
		Total	48	27 (56.5)	
Model 3	NCCN‐IPI ≤ 3	ΔSUV_max_ ≥ 86.02%	23	22 (95.70)	0.198
		ΔSUV_max_ < 86.02%	26	21 (80.80)	
		Total	49	43 (87.80)	
	NCCN‐IPI > 3	ΔSUV_max_ ≥ 86.02%	12	10 (83.30)	0.009
		ΔSUV_max_ < 86.02%	24	9 (37.50)	
		Total	36	19 (52.80)	
Total		Total	85	62 (72.90)	

NCCN‐IPI, National Comprehensive Cancer Network International Prognostic Index; PFS, progression‐free survival; SUV_max_, maximum standardized uptake value; TLG, total lesion glycolysis (subscripts 0 and 1 represent baseline and interim measures, respectively).

a Log‐rank test.

## 4 DISCUSSION

In the rituximab era, the risk stratification value of the IPI score has become weaker with increase in the curative rate, especially for the high‐intermediate and high‐risk groups.^2^ Therefore, more impactful prognostic tools are urgently needed. Herein, we mainly discuss the IPI‐related score system and PET/CT‐related parameters.

Some studies had revised the IPI score by adding new clinical prognostic factor(s),^18^ regrouping the original IPI score,^2^, ^19^ or specifically focusing on elderly patients (E‐IPI).^20^ The NCCN‐IPI was the most ideal one. By readjusting the age, LDH ratio, and extranodal disease, the NCCN‐IPI showed a better discrimination of patient outcomes (both overall survival and PFS) compared with the original IPI. However, some studies concluded that the NCCN‐IPI was not useful or that a PFS of 40‐60% still remained for the high‐risk group.^2^, ^3^, ^4^, ^5^, ^6^ In our study, K‐M survival analysis showed distinct differences among the four risk groups except the low and low‐intermediate groups. In the multivariate Cox regression analysis, the NCCN‐IPI scores categorized into four risk groups showed no significance, but the dichotomized NCCN‐IPI scores (low‐ and low‐intermediate vs. high‐ and high‐intermediate risk groups) significantly predicted PFS independently. The NCCN‐IPI is an optimal predictive tool owing to its convenience and repeatability. It can also be combined with interim parameters (ie SUV_max1_, TMTV_1_, and TLG_1_, or their variance ratios) to form a screening model for high‐risk patients.

On the other hand, some studies focused on filtering PET/CT‐related quantitative parameters for discriminating patients with a poor prognosis, and attempted some response‐adapted clinical trials. Baseline and interim (after 2‐4 cycles of chemotherapy) parameters (ie SUV_max_, TMTV, TLG, and their variance ratios) were studied, but obtained some controversial results.^9^, ^10^, ^11^, ^12^, ^13^

Because of its convenience and repeatability, SUV_max _has become the most commonly used PET/CT parameter, but its prognostic value also has not reached a consensus.^9^, ^10^, ^11^, ^12^, ^13^ According to mainstream opinions,^9^, ^10^ high baseline and interim SUV_max _measures indicate a poor outcome, reflecting a high proliferation of the tumor. In contrast, Gallicchio et al^11^ found that a higher SUV_max0_ was associated with better PFS. This also denied the predictive significance of TMTV_0_ and TLG_0_. Those authors surmised that patients with a high baseline metabolic activity usually respond right away to chemotherapy. Adams et al^12^ also argued that baseline SUV_max_, TMTV, and TLG values had no predictive significance. In that study, the median values of SUV_max0_, TMTV_0_, and TLG_0_ were used as the cutoff values, respectively, rather than the results of ROC curve analysis. In our study, SUV_max0_ showed no significance and the interim SUV_max _measure (%ΔSUV_max_ < 86.02) was statistically significant in the univariate Cox analysis, and %ΔSUV_max_ entered the multivariate model and presented predictive value independently in the multivariate model. The baseline SUV_max_ represented the metabolic and proliferative status, while the interim SUV_max_‐related parameters could assess the chemotherapeutic response and interim proliferative status of the tumor.

However, the SUV_max_ representing only one‐pixel point of the lesion could not reflect the condition of the whole lesion. In particular, for low‐uptake lesions, their uptake values were often overestimated as a result of background noise.^21^ In contrast, SUV_mean_ made up for the shortcomings of SUV_max_. Consequently, the volume parameter TLG, derived from SUV_mean_ and TMTV, may perform better in predicting the metabolic activity of the total lesion.

TLG and TMTV, which require a three‐dimensional delineation or segmentation of FDG‐avid lesions from PET/CT, could give a better evaluation of the tumor burden for patient risk stratification. Their volumes are usually measured by several different SUV_max_ thresholds: a fixed 41% SUV_max _threshold; an absolute threshold (>2.5 is commonly used); a method of liver SUV_mean_ plus 2 standard deviations (SDs) as a marginal threshold; and a visually adjusted variable SUV_max_ threshold. On the basis of the recommendation of the European Association of Nuclear Medicine guidelines^7^ and the research by Meignan et al^22^ we chose the fixed 41% SUV_max_ threshold owing to its better reproducibility and interobserver agreement. Because of the different patient ethnicities and measurement methods, the optimal cutoffs for TMTV_0_ and TLG_0_ varied from 70 to 850.3 cm^3^ 8, 13, 23, 24, 25, 26, 27 (TMTV_0_) and 826.5 to 4758 g 8, 13, 26, 27 (TLG_0_). Our optimal cutoffs for TMTV_0_and TLG_0_ were 80.74 cm^3^ and 1036.6 g, respectively, which were similar to the 70 cm^3^ and 826.5 g cutoff values used in the study of Zhou et al^13^ about Chinese patients. Although the 41% threshold method may give lower results compared with the other methods, the TMTV_0_ and TLG_0_ of our study showed significant prognostic value independently.

However, the fixed 41% SUV_max_ threshold does not always result in useful tumor definitions owing to noise, tracer uptake in homogeneities in the tumor and background, and sometimes a low tumor/background ratio. In our study, the optimal cutoffs of TMTV_1_ and TLG_1_ were skewed toward very low values (4.32 cm^3^ and 14.07 g, respectively); in particular, the cutoffs of %ΔTMTV and %ΔTLG were nearly 100% (99.22% and 99.86%, respectively), which were similar to those reported by Mikhaeel et al^8^ The excessive percentages restricted the clinical value of %ΔTMTV and %ΔTLG. Because of the low interim SUV_max_ measures, the fixed 41% SUV_max_ threshold method may cause underestimated values when delineating VOIs. Although TMTV_1_ and TLG_1_ showed significant value for predicting PFS in the Cox regression analysis, we surmise that they could have better performance if the liver SUV_mean_ plus 2SDs or absolute threshold (SUV > 2.5) methods were used instead to delineate the interim tumor lesions. More studies are needed to confirm if this is true.

Several studies^13^, ^28^, ^29^, ^30^ have reported TLG as being a more powerful predictor of outcomes for patients with DLBCL. Zhou et al,^13^ Esfahani et al,^28^ and Ceriani et al^29^ surmised that TLG was the only independent predictor, rather than TMTV and SUV_max_. Whereas other four studies^8^, ^24^, ^25^, ^31^ analyzing baseline TMTV only concluded that TMTV was the independent predictor for PFS (Table S4). Xie et al analyzed both of baseline TMTV and TLG and concluded that both were independent predictors. However, in our study, we found a strong correlation between TMTV and TLG, meaning that when TMTV and TLG were both included into the model equation, the more powerful one would kick out the other from the regression model. In the former studies mentioned above, the *P*‐values of their univariate analyses for TMTV and TLG were usually less than 0.001, and they had very similar K‐M survival curve results, respectively. However, all of those studies forcibly combined TMTV and TLG into the Cox model equation, which may lead to the mistaken elimination of TMTV or TLG. Consequently, we tried not to incorporate the correlated variables into the Cox regression analysis model equation simultaneously.

We found that the baseline parameters TMTV_0_ and TLG_0 _had 91.3% sensitivity, respectively (Table 2), which could help discriminate the majority of patients with poor outcomes. The interim parameters TMTV_1_ and TLG_1_ showed 87.1% and 85.5% specificity, respectively, helping to distinguish even more patients with a high risk of relapse or progression from those with baseline high risk. The %ΔSUV_max_ (derived from baseline and interim data with 87% sensitivity) could also re‐discriminate patients with a high relapse risk after 2‐4 cycles of R‐CHOP chemotherapy. Although TLG_0_, SUV_max1_, TLG_1_, %ΔSUV_max_, %ΔTLG, and NCCN‐IPI all showed significance in the K‐M survival analysis, only TLG_0_, TLG_1_, and %ΔSUV_max _were entered into the multivariate model, where upon only TLG_0_ and %ΔSUV_max_ demonstrated predictive value independently. We combined the baseline and interim variables into three prognostic models. Model 2, consisting of TLG_0_ and %ΔSUV_max_, was superior in screening high‐risk patients, where the group of TLG_0_ > 1036.6 cm^3^ and %ΔSUV_max_ < 86.02% picked out 19 (82.6%) of the relapse or progression events, whereas only four events were omitted in the other three groups. Model 2 showed a better prognostic ability than model 1 (TLG_0_ + TLG_1_) and model 3 (dichotomized NCCN‐IPI + %ΔSUV_max_). Patients with low baseline TLG results received a three‐year PFS of approximately 94.6% and had no relationship with %ΔSUV_max_. For patients with high baseline results, whether %ΔSUV_max_ was below or above 86.2% would predict their outcomes (three‐year PFS: 32.1% vs. 90.0%).

Adams and Kwee^6^ reviewed and conducted a meta‐analysis of nine studies on the prognostic value of interim PET/CT in R‐CHOP‐treated DLBCL. They found that the prognostic value was homogeneously suboptimal across the studies, and it was not consistently proven to surpass the prognostic potential of the IPI. There is a lack of studies comparing interim PET/CT parameters with the newly developed NCCN‐IPI. Our study compared PET/CT parameters with NCCN‐IPI by using Cox regression analysis. The NCCN‐IPI scores categorized into four risk groups showed no significance in the multivariate regression model and were therefore considered unusable for this model (Table 3). However, the dichotomized NCCN‐IPIs were entered into the model and showed independent predictive value (HR = 3.0, 95% CI 1.08‐8.33, *P*‐value = 0.035, in model of TLG; HR = 3.61, 95% CI 1.36‐9.56, *P‐*value = 0.01, in model of TMTV), similar to TLG and TMTV. Thus, we combined the dichotomized NCCN‐IPI with ΔSUV_max_ into model 3, but the result was not as good as that obtained with model 2.

From the results of our research, the baseline variables, that is, TMTV_0_, TLG_0_ and dichotomized NCCN‐IPI, and the interim variables TMTV_1_ and %ΔSUV_max_, presented independent prognostic value for PFS, and the model consisting of the baseline and interim parameters (model 2 [TLG_0_ + %ΔSUV_max_]) also presented superior screening ability. The repeatability and effectiveness of our results still need to be validated by more studies. However, unifying the various delineating methods must be a priority, which need more studies to make a consensus about the method of threshold.

## 5 CONCLUSIONS

The results of our study showed the independent prognostic abilities of TLG_0_ and ΔSUV_max_. When replacing TLG with TMTV measures, TMTV_0_ and TMTV_1_ also showed independent prognostic value in the multivariate Cox regression model. Dichotomized NCCN‐IPI also got the same result. Model 2 comprising TLG_0_ and %ΔSUV_max_ picked out 19 (82.6%) of the relapse or progression events, demonstrating that a model combining baseline and interim parameters can be a powerful prognostic tool. Generally speaking, the baseline quantitative parameters of PET/CT (TMTV_0_ and TLG_0_) had the best predictive ability. The %ΔSUV_max_ could also help with further screening. However, the method of a fixed 41% threshold was not satisfactory owing to a lower lesion/background ratio of SUV_max_ in delineating and calculating the interim volume parameters (ie TMTV_1_ and TLG_1_), which may cause these values to be underestimated. Consequently, we thought that maybe the methods of liver SUV_mean_ plus 2SDs or absolute threshold (SUV > 2.5) could be used for detecting interim volume parameters after the method of a fixed 41% threshold has delineated and calculated the baseline volume parameters. Whether these tools could be used for driving response‐adapted therapy still needs further validation in clinical trials.

## CONFLICT OF INTEREST

None of the authors have any conflict of interest to disclose.
