# Supplementary material for: A better prediction of progression‐free survival in diffuse large B‐cell lymphoma by a prognostic model consisting of baseline TLG and %ΔSUVmax
Source: Cancer Med. 2019 Jul 25;8(11):5137–47. doi: 10.1002/cam4.2284 (PMC6718622; doi:10.1002/cam4.2284)
Supplement: Supplementary file 1 [file CAM4-8-5137-s001.docx]

***Supporting information***

Supplement 1. Risk factors of clinical and quantitative PET/CT parameters (replacing TLG with TMTV) for PFS analyzed by univariate and multivariate Cox regression analyses

| Covariate | Univariate analyses | | | Multivariate analyses | | |
| --- | --- | --- | --- | --- | --- | --- |
|  | HR | 95%CI | *P*-value | HR | 95%CI | *P*-value |
| NCCN-IPI risk groups | - | - | 0.000 | - | - | - |
| Low | - | - | - | - | - | - |
| Low-intermediate | 1.79 | 0.21, 15.36 | 0.59 | - | - | - |
| High-intermediate | 5.72 | 0.73, 45.2 | 0.098 | - | - | - |
| High | 17.49 | 2.17, 140.99 | 0.007 | - | - | - |
| TMTV_0_ (>80.74) | 10.32 | 2.42, 44.084 | 0.002 | 8.22 | 1.86-36.24 | 0.005 |
| TLG_0_ (>1036.61) | 10.39 | 2.43, 44.39 | 0.002 | - | - | - |
| TMTV_1_ (>4.32) | 6.93 | 2.97, 16.17 | 0.000 | 2.96 | 1.16-7.54 | 0.023 |
| TLG_1_ (>14.07) | 6.25 | 2.68, 14.56 | 0.000 | - | - | - |
| %ΔSUV_max_ (<86.02) | 5.60 | 1.66, 18.88 | 0.005 | 3.73 | 1.00-13.90 | 0.05 |

HR, hazards ratio; CI, confidence interval; NCCN-IPI, National Comprehensive Cancer Network International Prognostic Index; SUV_max_, maximum standardized uptake value; MTV, metabolic tumor volume; TLG, total lesion glycolysis; PFS, progression-free survival. The subscripts 0 and 1 represent baseline and interim measures, respectively. The TLG variables were not included in the multivariate model owing to their correlation with the TMTV variables.

Supplement 2. Univariate and multivariate Cox regression analyses of dichotomized NCCN-IPI and quantitative PET/CT parameters (replacing TLG with TMTV) for PFS

| Covariate | Univariate analyses | | | Multivariate analyses | | |
| --- | --- | --- | --- | --- | --- | --- |
|  | HR | 95%CI | *P* | HR | 95%CI | *P* |
| Dichotomized NCCN-IPI | 5.23 | 2.058-13.31 | 0.001 | 3.61 | 1.36-9.56 | 0.01 |
| TMTV_0_ (>80.74) | 10.32 | 2.42, 44.084 | 0.002 | 4.73 | 1.047-21.41 | 0.043 |
| TLG_0_ (>1036.61) | 10.39 | 2.43, 44.39 | 0.002 | - | - | - |
| TMTV_1_ (>4.32) | 6.93 | 2.97, 16.17 | 0.000 | 5.52 | 2.26-13.44 | 0.000 |
| TLG_1_ (>14.07) | 6.25 | 2.68, 14.56 | 0.000 | - | - | - |
| %ΔSUV_max_ (<86.02) | 5.60 | 1.66, 18.88 | 0.005 |  |  |  |

The TLG variables were not included in the multivariate model owing to their correlation with the TMTV variables. NCCN-IPI was dichotomized into low (low and low-intermediate) and high (high and high-intermediate) risk groups.

Supplement 3. Univariate and multivariate Cox regression analyses of dichotomized NCCN-IPI and quantitative PET/CT parameters (replacing TMTV with TLG) for PFS

| Covariate | Univariate analyses | | | Multivariate analyses | | |
| --- | --- | --- | --- | --- | --- | --- |
|  | HR | 95%CI | *P* | HR | 95%CI | *P* |
| Dichotomized NCCN-IPI | 5.23 | 2.058-13.31 | 0.001 | 3.00 | 1.08-8.33 | 0.035 |
| TMTV_0_ (>80.74) | 10.32 | 2.42, 44.084 | 0.002 | - | - | - |
| TLG_0_ (>1036.61) | 10.39 | 2.43, 44.39 | 0.002 | 5.84 | 1.20-28.44 | 0.029 |
| TMTV_1_ (>4.32) | 6.93 | 2.97, 16.17 | 0.000 | - | - | - |
| TLG_1_ (>14.07) | 6.25 | 2.68, 14.56 | 0.000 | 3.08 | 1.15-8.25 | 0.025 |
| %ΔSUV_max_ (<86.02) | 5.60 | 1.66, 18.88 | 0.005 | 3.80 | 0.98-14.77 | 0.054 |

The TMTV variables were not included in the multivariate model owing to their correlation with the TLG variables. NCCN-IPI was dichotomized into low (low and low-intermediate) and high (high and high-intermediate) risk groups.

Supplement 4. Summary of previous studies about PET/CT parameters for predicting PFS.

| Authors (year) | Simple size | Follow-up (months) | Methods of threshold* | Baseline and interim PET/CT parameters predicting for PFS (significantly: yes or not) | | | | |
| --- | --- | --- | --- | --- | --- | --- | --- | --- |
|  |  |  |  | PFS | Baseline parameters | | Interim parameters | |
|  |  |  |  |  | Yes | Not | Yes | Not |
| Park S. et al.^1^ 2012 | 100 | 21 | SUV of mediastinal blood pool | 2-year | SUV_sum_ | SUV_max,_  TLG_sum_ | SUV_max,_  SUV_sum_ | TLG_sum,_ %ΔSUV_max,_  %ΔSUV_sum,_  %ΔTLG_sum_ |
| Casasnovas et al.^2^ 2012 | 121 | 28 | 41% of SUV_max,_ | 2-year | TMTV | — | — | — |
| Sasanelli et al. ^5^ 2014 | 114 | 39 | 41% of the SUV _max,_ | 3-year | TMTV | aaIPI,  Bulk≥10cm | — | — |
| Ceriani et al.^6^ 2015 | 103 | 36 | 25% of the SUV _max,_ | 5-year | TLG | — | — | — |
| Adams et al. 2015 | 73 | 33 | 40% of SUV_max_ | NA | NCCN-IPI | SUV_max_ TLG TMTV | — | — |
| Zhou et al^3^ 2016 | 91 | 30 | Liver SUVmean plus 3SD | 5-year | TLG | TMTV,  NCCN-IPI | — | — |
| Mikhaeel et al.^4^ 2016 | 147 | 45 | SUV > 2.5 | 5-year | TMTV† | IPI,  SUV _max,_ |  | DS, TMTV, SUV_max_  ΔSUV _max,_ |
| Xie MX et al.^7^ 2016 | 60 | 17 | Liver SUVmean plus 2SD | NA | TLG,  TMTV | SUV _max,_ | — | — |
| Shagera et al.^8^ 2019 | 103 | 34 | SUV > 2.5 | 3-year | TMTV†,  NCCN-IPI | — | — | — |

*methods of threshold for delineating the region of interest around the focus lesions to calculate the results of MTV and TLG.

† TLG was not used for the multivariate analysis due to the strong correlation ship between TMTV and TLG. ΔTLG and ΔTMTV were also not included into multivariate analysis.

DS, Deauville Score

1. Park S, Moon SH, Park LC, et al. The impact of baseline and interim PET/CT parameters on clinical outcome in patients with diffuse large B cell lymphoma. *Am J Hematol* 2012;87:937-940.

2. Casasnovas RO, Sasanelli M, Berriolo-Riedinger A, et al. Baseline Metabolic Tumor Volume Is Predictive of Patient Outcome in Diffuse Large B Cell Lymphoma. *Blood* 2012;120.

3. Zhou M, Chen Y, Huang H, et al. Prognostic value of total lesion glycolysis of baseline 18F-fluorodeoxyglucose positron emission tomography/computed tomography in diffuse large B-cell lymphoma. *Oncotarget* 2016;7:83544-83553.

4. Mikhaeel NG, Smith D, Dunn JT, et al. Combination of baseline metabolic tumour volume and early response on PET/CT improves progression-free survival prediction in DLBCL. *Eur J Nucl Med Mol Imaging* 2016;43:1209-1219.

5. Sasanelli M, Meignan M, Haioun C, et al. Pretherapy metabolic tumour volume is an independent predictor of outcome in patients with diffuse large B-cell lymphoma. *Eur J Nucl Med Mol Imaging* 2014;41:2017-2022.

6. Ceriani L, Martelli M, Zinzani PL, et al. Utility of baseline 18FDG-PET/CT functional parameters in defining prognosis of primary mediastinal (thymic) large B-cell lymphoma. *Blood* 2015;126:950-956.

7. Xie MX, Zhai WH, Cheng SY, et al. Predictive value of F-18 FDG PET/CT quantization parameters for progression-free survival in patients with diffuse large B-cell lymphoma. *Hematology* 2016;21:99-105.

8. Shagera QA, Cheon GJ, Koh Y, et al. Prognostic value of metabolic tumour volume on baseline (18)F-FDG PET/CT in addition to NCCN-IPI in patients with diffuse large B-cell lymphoma: further stratification of the group with a high-risk NCCN-IPI. *Eur J Nucl Med Mol Imaging* 2019.
